# Esophageal cancer: trends in incidence and mortality in China from 2005 to 2015

**DOI:** 10.1002/cam4.3647

**Published:** 2021-02-16

**Authors:** Feifan He, Junyi Wang, Li Liu, Xiaoyue Qin, Zhanyong Wan, Wei Li, Zhiguang Ping

**Affiliations:** ^1^ College of Public Health, Zhengzhou University Zhengzhou Henan China; ^2^ Basic Medical School, Zhengzhou University Zhengzhou Henan China

**Keywords:** China, Esophageal cancer, incidence or morbidity, mortality, trend

## Abstract

**Background:**

The long‐term trend analysis of esophageal cancer is rarely reported in China. Our purpose is to analyze the incidence and mortality trends of esophageal cancer in China from 2005 to 2015.

**Method:**

Based on *the* data *in the annual report of the China Cancer Registry,* a comprehensive analysis of esophageal cancer cases and deaths from 2005 to 2015 was carried out. The incidence and mortality of esophageal cancer are stratified by gender and region (urban or rural). Long‐term trend analysis was conducted using Joinpoint regression model.

**Result:**

In China, the age‐standardized incidence rates by the world population declined from 13.84/10^5^ in 2005 to 11.64/10^5^ in 2015. Annual percent changes were 3.4% (95% CI: 0.6%, 6.3%) in the period 2005‐2011, −7.4% (95% CI: −10.1%, −4.7%) in the period 2011‐2015, respectively. The age‐standardized mortality rates declined from 10.86/10^5^ in 2005 to 8.57/10^5^ in 2015. And the average annual percent change was −4.1% (95% CI: −6.7%, −1.5%). The incidence and mortality of esophageal cancer in men are higher than those in women, and the incidence and mortality of esophageal cancer in rural areas are much higher than those in urban areas.

**Conclusion:**

In China, the incidence of esophageal cancer first increased and then decreased during 2005‐2015, while the mortality rate has been declining.

## Graphical abstract

In China, the incidence of esophageal cancer increased first and then decreased during 2005‐2015, and the mortality rate has been declining throughout the period.

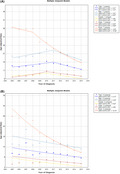

## 1 INTRODUCTION

Globally, esophageal cancer is one of the most frequently reported cancer types and a common cause of death.^1^ In China, the incidence of esophageal cancer in 2015 ranked sixth and the mortality rate ranked fourth.^2^ According to the GLOBOCAN 2018 report of the International Agency for Research on Cancer (IARC), the number of global esophageal cancer cases is 572,034, accounting for 3.2% of the total number of new cases; the death toll is 508,585, accounting for 5.3% of all cancer deaths. The age‐standardized incidence rate (ASIR) of men is higher than that of women, with a ratio of approximately 2.7:1. When assessing the age‐standardized mortality rate (ASMR), gender differences were also observed.^3^

Some studies on the incidence and mortality of esophageal cancer have been conducted worldwide. According to data from the Japan Cancer Control and Information Service Center, Lin Yingsong’s research shows that the ASIR for esophageal cancer increased from 8.3/10^5^ in 1975 to 11.7/10^5^ in 2006.^4^ On the contrary, a decline in esophageal cancer has been found in Korea. ASIR dropped from 4.06/10^5^ in 1999 to 2.91/10^5^ in 2013, with an annual percent change (APC) of −2.2% ( *p* < 0.001).^5^ A similar trend in esophageal cancer has been found in American women; from 2001 to 2015, the APC was −1.41%.^6^ In addition, using data from the Korean Statistical Information Service (KOSIS) database, a time trend analysis in South Korea shows that the trends in esophageal cancer mortality are similar in men and women. For men, the mortality rate increased from 1983 to 1993, the APC was 4.14%, and it decreased from 1993 to 2002, the APC was −2.34%, and then, with a sharp decline from 2002 to 2012, it was −5.12%. For women, there was no significant trend from 1983 to 1995. Since then, the mortality rate has decreased, with APC of −6.30%.^7^

However, in China, there are few epidemiological studies on the long‐term trend of esophageal cancer. Most existing morbidity and mortality studies are limited to one year or a short period of time or in specific areas. Based *on* data from the *annual report of the China Cancer Registry*, our study describes the incidence and mortality of esophageal cancer in China from 2005 to 2015. The number of new cancer cases and deaths, corresponding morbidity, mortality, and age‐standardized rate (ASR) are reported. In addition, we analyzed the incidence and mortality trends of esophageal cancer during the entire study period, as well as gender and regional diversity. 

## 2 MATERIALS AND METHODS

The National Central Cancer Registry of China (NCCR) is responsible for the collection, evaluation and release of cancer data according to the local population cancer registry. Cancer data was collected from clinics in rural areas, local hospitals, health insurance databases, death monitoring databases and cooperative medical insurance databases. Then, checking the data according to the “Chinese Cancer Registry Guidelines” and the “Five Continent Stage IX Cancer Incidence Rate” standards of the International Agency for Research on Cancer (IARC)/International Association of Cancer Registry (IACR). From 2005 to 2015, the number of cancer registries increased from 45 to 501, the population covered increased from 69 to 388 million, and the proportion of the national population increased from 5.31% to 28.22%. After quality assessment, the number of cancer registries included in the database has increased from 34 to 388, the population covered has increased from 55 to 321 million, and the proportion of the total population of the country has increased from 4.20% to 23.35%. In 2015, all 31 provinces, autonomous regions, municipalities directly under the Central Government and Xinjiang Production and Construction Corps in mainland China established a cancer registration framework, and comprehensively reported cancer incidence and mortality.^2^

The data on esophageal cancer includes new cases, deaths, morbidity, mortality and ASR from 2005 to 2015, extracted from the *annual report of the Chinese Cancer Registry*. The cases of esophageal cancer (C15) were determined by the tenth revision of the International Statistical Classification of Diseases and Related Health Problems (ICD‐10). Included data between January 1 and December 31 of the year, all cases were diagnosed with esophageal cancer or died of esophageal cancer.

The incidence and mortality of esophageal cancer are stratified by gender (male or female) and region (urban or rural). The ASIR and ASMR of the Chinese population are standardized by the Chinese population structure of 1982 (2005‐2009) or 2000 (2010‐2015). Segi's world population structure standardizes the ASIR and ASMR of the world population. All rates are expressed per 100,000 person‐years. Connection point regression analysis, by linking several different linear segments on the logarithmic scale of the connection point to describe the change of the result trend.^8^ The morbidity and mortality trends of esophageal cancer from 2005 to 2015 were described. Using the joint point regression program (version 4.7.0.0) to calculate the average annual rate of change (AAPC), APC and its 95% confidence interval (95% CI). In addition, the number and location of the connection points and the corresponding *P* value are determined by permutation tests. The significance level α of both sides is taken as 0.05.

## 3 RESULTS

### 3.1 Incidence and mortality of esophageal cancer

By 2015, China newly diagnosed cases of esophageal cancer was 61 732 (44,067 for males and 17,667 for females), the crude incidence rate of 19.24 / 10 ^5^, new cancer cases accounted for 6.69%. The ASIRs of the Chinese population and the world population are 11.50/10 ^5^ and 11.64/10 ^5^ respectively, ranking sixth in the incidence of all cancers. Among them, the rough hair rate of men is 27.07/10 ^5^, accounting for 8.64% of new cancer cases in men. The ASIRs of the Chinese population and the world population are 16.96/10 ^5^ and 17.19/10 ^5^ respectively. The incidence of brutality among women is 11.17/10 ^5^, accounting for 4.28% of new cancer cases in women. The ASIRs of the Chinese population and the world population are 6.21/10 ^5^ and 6.24/10 ^5^ respectively. From 2005 to 2015, the incidence of esophageal cancer first increased, and then declined by gender in the general population and subgroups, reaching a peak in 2010. In addition, the number of new cases in men in 2015 was significantly higher than that in women, with a ratio of approximately 2.5:1 (Table 1).

**Table 1:** The incidence of esophageal cancer in registered areas in China from 2005 to 2015

Year	Gender	Number of cases	Crude oil rate (1/10 ^5^ )	Proportion(%)	ASIR China (1/10 ^5^ )	ASIR World (1/10 ^5^ )
2005	All	10 738	19.55	7.57	10.24	13.84
	male	7321	26.31	9.19	14.55	19.65
	Female	3417	12.61	5.49	6.13	8.39
2006	All	11 195	18.79	6.87	9.68	13.11
	male	7653	25.50	8.39	13.83	18.77
	Female	3542	11.99	4.93	5.75	7.83
2007	All	11 877	19.86	7.19	10.14	13.71
	male	8115	26.85	8.80	14.39	19.46
	Female	3762	12.72	5.16	6.05	8.26
2008	All	13 792	20.85	6.97	9.88	13.54
	male	9556	28.66	8.68	14.26	19.55
	Female	4236	12.92	4.83	5.69	7.87
2009	All	18 924	22.14	7.74	10.88	14.81
	male	13 161	30.44	9.57	15.62	21.27
	Female	5763	13.64	5.39	6.27	8.59
2010	All	30 364	24.36	8.69	16.19	16.48
	male	21 082	33.42	10.62	23.23	23.68
	Female	9282	15.07	6.15	9.44	9.57
2011	All	33 339	22.87	8.07	14.95	15.19
	male	23 549	31.98	10.10	21.79	22.18
	Female	9790	13.58	5.45	8.37	8.46
2012	All	44 967	22.70	8.09	14.66	14.86
	male	31 733	31.62	10.16	21.35	21.66
	Female	13 234	13.55	5.43	8.23	8.30
2013	All	50 197	22.16	7.79	13.92	14.09
	male	35 414	30.83	9.82	20.25	20.55
	Female	14 783	13.24	5.21	7.79	7.84
2014	All	58 396	20.26	7.08	12.49	12.64
	male	41 755	28.56	9.11	18.41	18.69
	Female	16 641	11.72	4.54	6.74	6.77
2015	All	61 734	19.24	6.69	11.50	11.64
	male	44 067	27.07	8.64	16.94	17.19
	Female	17 667	11.17	4.28	6.21	6.24

Note

The data comes from the *annual report of the China Cancer Registry*.

Abbreviations: ASIR China, age‐standardized incidence rate by Chinese population; ASIR World, age‐standardized incidence rate by world population.

2015, esophageal cancer deaths was 47 373 (34,262 for males and 13,111 for females), the crude mortality rate of 14.76 / 10 ^5^, accounting for 8.39 percent of the total cancer deaths. The ASMR of the Chinese population and the world population are 8.54/10 ^5^ and 8.57/10 ^5^ respectively, ranking fourth among all cancer death rates. Among them, the crude death rate for men was 21.05/10 ^5^, accounting for 8.64% of the total deaths from cancer in men. The ASMRs of the Chinese population and the world population are 12.92/10 ^5^ and 13.00/10 ^5^ respectively. The crude death rate for women is 8.29/10 ^5^, accounting for 6.34% of the total deaths from cancer in women. The ASMRs of the Chinese population and the world population are 4.34/10 ^5^ and 4.31/10 ^5^ respectively. During the period 2005‐2015, the mortality rate gradually declined. Similar to the morbidity rate, the mortality rate of men is also higher than that of women, with a mortality rate of approximately 2.6:1 (Table 2).

**Table 2:** 2005‐2015 Mortality of Esophageal Cancer in Registered Areas of China

Year	Gender	Number of cases	Crude oil rate (1/10 ^5^ )	Proportion(%)	ASMR China (1/10 ^5^ )	ASMR World (1/10 ^5^ )
2005	All	8669	15.78	9.34	7.91	10.86
	Male	5843	21.00	10.15	11.27	15.46
	Female	2826	10.43	8.02	4.77	6.61
2006	All	9092	15.26	8.69	7.46	10.30
	Male	6216	20.71	9.52	10.82	14.97
	Female	2876	9.73	7.30	4.33	6.04
2007	All	9450	15.80	8.92	7.58	10.47
	Male	6522	21.58	9.84	11.10	15.32
	Female	2928	9.90	7.38	4.25	5.96
2008	All	10 741	16.24	8.79	7.34	10.11
	Male	7482	22.44	9.84	10.84	14.90
	Female	3259	9.94	7.07	4.02	5.63
2009	All	14 337	16.77	9.29	7.75	10.76
	Male	10 067	23.29	10.39	11.42	15.86
	Female	4270	10.11	7.44	4.22	5.96
2010	All	22 292	17.88	10.05	11.51	11.59
	Male	15 422	24.45	11.08	16.76	16.93
	Female	6870	11.16	8.33	6.56	6.56
2011	All	25 282	17.35	9.72	11.03	11.13
	Male	17 836	24.22	10.86	16.29	16.48
	Female	7446	10.33	7.75	6.06	6.09
2012	All	33 258	16.79	9.63	10.54	10.59
	Male	23 433	23.35	10.78	15.56	15.69
	Female	9825	10.06	7.67	5.77	5.74
2013	All	37 690	16.64	9.44	10.15	10.18
	Male	26 457	23.03	10.52	14.93	15.02
	Female	11 233	10.06	7.61	5.59	5.55
2014	All	43 383	15.05	8.67	9.01	9.05
	Male	31 261	21.38	9.89	13.57	13.65
	Female	12 122	8.53	6.58	4.65	4.62
2015	All	47 373	14.76	8.39	8.54	8.57
	Male	34 262	21.05	9.57	12.92	13.00
	Female	13 111	8.29	6.34	4.34	4.31

Note

The data comes from the *annual report of the China Cancer Registry* .

Abbreviations: ASMR China, age‐standardized incidence rate by Chinese population; ASMR World, age‐standardized incidence rate by world population.

**Figure 1:**
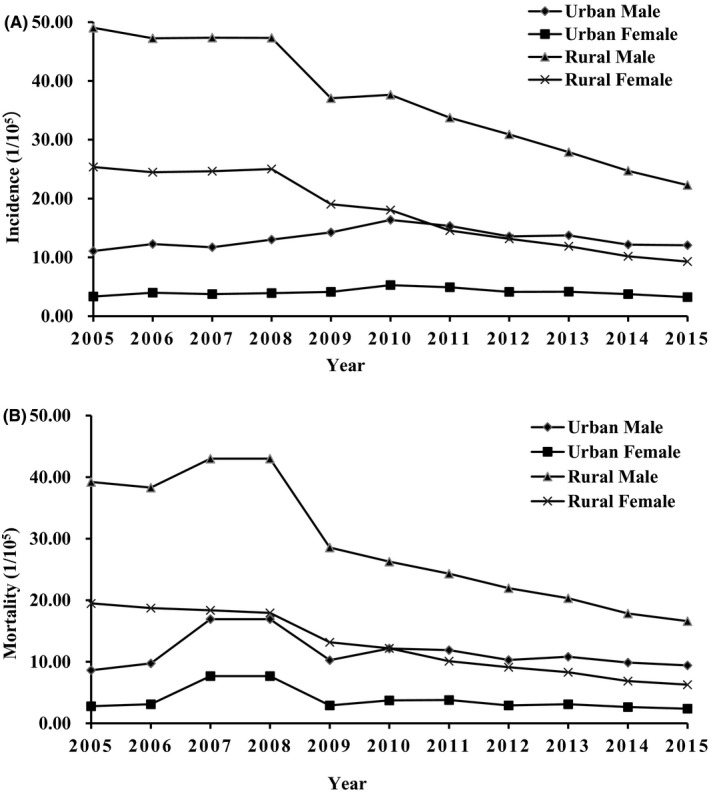
The ASIR world (A) and ASMR world (B) of esophageal cancer in urban and rural areas by gender from 2005 to 2015.

Grouped by gender and region, the ASIR order of esophageal cancer from high to low is rural males, rural females, urban males, and urban females. Unlike other subgroups, the incidence of rural males is decreasing year by year. As mentioned above, the incidence of men is higher than that of women. In addition, the incidence of patients in rural areas is higher than that in urban areas. In recent years, the incidence in rural areas has shown a downward trend, while urban areas have remained unchanged (Figure 1A). When assessing ASMR, a similar shift was observed (Figure 1B).

### 3.2 Time trend of esophageal cancer incidence and mortality

**Figure 2:**
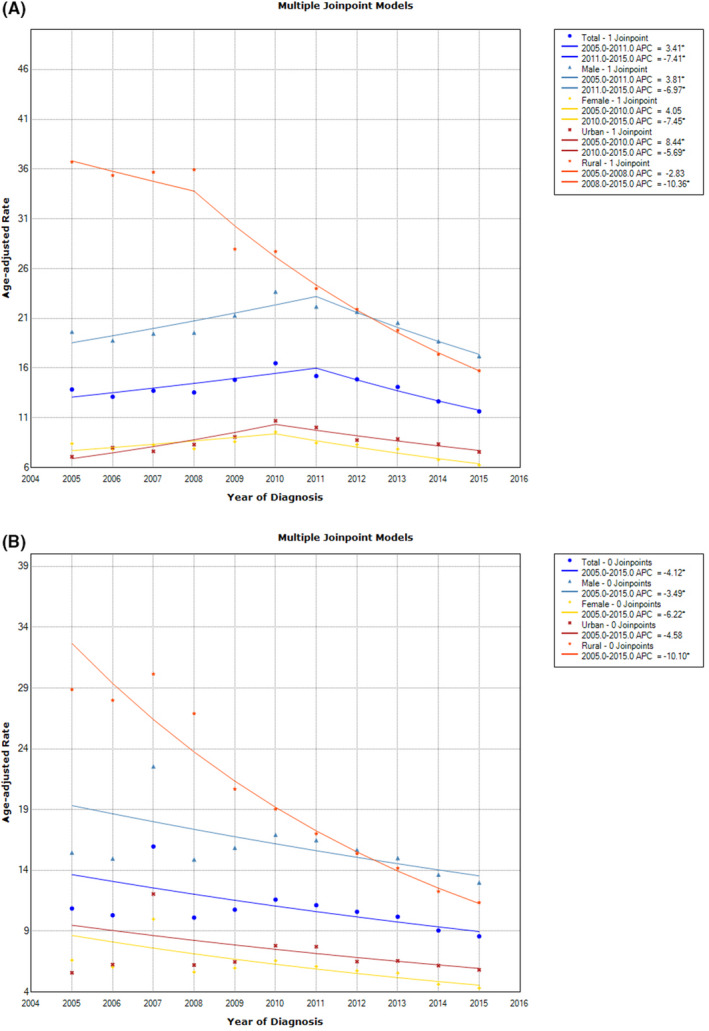
The trend of esophageal cancer during 2005‐2015; (A) Stratification by gender and region; (B) Dividing regions by gender and mortality.

From 2005 to 2011, the incidence of esophageal cancer gradually increased when the APC was 3.4% (95% CI: 0.6%, 6.3%), but sharply decreased at an APC of −7.4% (95% CI: −10.1%, −4.7%) during 2011‐2015. In addition to the different connection points, similar esophageal cancer trends have also been observed in men and women. From 2005 to 2011, the incidence of men increased, APC was 3.8% (95% CI: 1.1%, 6.6%); from 2011 to 2015, the incidence decreased, APC was −7.0% (95% CI: −9.4%, −4.4%). For women, this connection point appeared in 2010, and there was no statistically significant trend from 2005 to 2010. Since then, the incidence of APC has decreased by −7.5% (95% CI: −9.8%, −5.0%). After stratified by region, the incidence rate in urban areas increased with an APC of 8.4% from 2005 to 2010 (95% CI: 4.0%, 13.0%). From 2010 to 2015, the incidence rate decreased at an APC of −5.7% (95% CI: −8.0%, −3.3%). In rural areas, the incidence rate decreased throughout the study period, AAPC was −8.2% (95% CI: −10.0%, −6.3%) (Table 3, Figure 2A).

**Table 3:** Trends in the incidence and mortality of esophageal cancer in China from 2005 to 2015 by gender.

Variable	Incidence			Mortality
	Year	Armored personnel carrier	IAEA	IAEA
Total	2005–2011	3.4^A kind^ (0.6, 6.3)	−1.1 (−2.7, 0.6)	−4.1^A kind^ (−6.7, −1.5)
	2011–2015	−7.4^A kind^ (−10.1, −4.7)		
Gender				
Male	2005–2011	3.8^A kind^ (1.1, 6.6)	−0.6 (−2.2, 0.9)	−3.5^A kind^ (−5.9, −1.1)
	2011–2015	−7.0^A kind^ (−9.4, −4.4)		
Female	2005–2010	4.1 (‐0.6, 8.9)	−1.9 (−3.9, 0.2)	−6.2^A kind^ (−9.3, −3.0)
	2010–2015	−7.5^A kind^ (−9.8, −5.0)		
Area				
Urban area	2005–2010	8.4^A kind^ (4.0, 13.0)	1.1 (‐0.8, 3.1)	−4.6 (−9.1, 0.1)
	2010–2015	−5.7^A kind^ (−8.0, −3.3)		
Rural	2005–2008	−2.8 (−10.1, 5.1)	−8.2^A kind^ (−10.0, −6.3)	−10.1^A kind^ (−11.2, −9.0)
	2008–2015	−10.4^A kind^ (‐11.4, ‐9.3)		

Abbreviations: AAPC, average annual percentage change; APC, annual percentage change.

For mortality, Joinpoint regression analysis showed that the AAPC of the total population from 2005 to 2015 was −4.1% (95% CI: −6.7, −1.5). In terms of subgroups, males (AAPC: −3.5%, 95% CI: −5.9%, −1.1%), females (AAPC: −6.2%, 95% CI: −9.3%, −3.0%) and rural areas (AAPC: −10.1%, 95% CI: −11.2%, −9.0%). However, the AAPC in urban areas was not statistically significant (Table 3, Figure 2B).

## 4 DISCUSSION

From 2005 to 2015, the incidence of esophageal cancer in China increased first (2005‐2011: APC: 3.4%) and then decreased (2011‐2015: APC: ‐7.4%), while the mortality rate decreased year by year. Unlike our results, in other countries, both morbidity and mortality are on the rise. In Brazil, the incidence of esophageal cancer from 2005 9.1 / 10 ^5^ increased to 2015 years of 12.1 / 10 ^5^ .^9^ In Serbia, during the 1991‐2015 study period, the APC of male esophageal cancer mortality was 0.9% (95% CI: 0.3%, 1.4%) and 0.4% (95% CI: ‐0.6%, 1.4%) for women.^10^ Smoking is one of the established risk factors for esophageal cancer.^11^ At the same time, China has a relatively widespread smoking epidemic and second‐hand smoke exposure.^12^ The obvious trend of esophageal cancer may be attributed to the long‐term efforts of national public health measures to ban indoor smoking in the past few decades. On November 10, 2003, China officially became the 77th country to sign the *Framework Convention on Tobacco Control*. In 2006, the Seventeenth Meeting of the Standing Committee of the Tenth National People's Congress formally approved the above convention, prohibiting the use of vending machines to control tobacco.^13^ In 2011, the Chinese government passed the *"Detailed Rules for the Implementation of the Regulations on Sanitation Administration in Public Places*.*"* And bansmoking took effect on May 1 of the same year. It is clearly prohibited to smoke in indoor public places.^14^ The relevant data show that compared with 2010, the proportion of smoking and second‐hand smoke exposure in public places such as restaurants and government buildings has dropped significantly in 2015.^15^ In addition, in 2008, the Chinese government invested approximately RMB 400 billion in health, culture and education.^16^ This has improved the Chinese people's awareness of disease prevention and medical care, and further reduced the incidence of esophageal cancer. There is no doubt that stratified analysis based on some other important variables, such as histological type, tumor stage and tumor size, will help us better understand the current results. However, the inability to obtain detailed information about the clinicopathological characteristics of esophageal cancer is also a limitation of our study, which is inherent in the reported registration data.

In addition, esophageal cancer screening may contribute to the uninterrupted decline in mortality (AAPC: ‐4.1%). Facts have proved that endoscopic screening using iodine staining can effectively improve the 5‐year survival rate and reduce mortality of patients with esophageal cancer. This is currently the best early screening method.^17^ The central government expanded the scope of subsidies to local public health funds in 2005, including the screening, early diagnosis and treatment of esophageal cancer implemented in 2006.^18^ The tumor stage at diagnosis was associated with the prognosis of esophagus cancer, and early detection could effectively reduce its mortality.^19^ A long‐term follow‐up study on the surgical outcome of Chinese patients with esophageal cancer showed that the 5‐year survival rate for early diagnosis exceeds 85%, and the 5‐year survival rate for late diagnosis is less than 10%.^20^

In terms of gender‐specific rates, as shown in Table 1 and 2, the incidence and mortality were much higher in males than in females at approximately two to three times greater. This difference was also found in other studies, which supports our results. Coincidentally, similar phenomena were found in two different subtypes of esophageal cancer. Esophageal squamous cell carcinoma (ESCC) is the main histological type in China, and males dominate, while esophageal adenocarcinoma (EAC) is common in most western countries (such as the United States, the United Kingdom and Wales) and is more common in women. For EAC, risk factors include increased body mass index and gastroesophageal reflux disease. However, smoking and drinking are the main risk factors for ESCC.^1^, ^21^ Therefore, research on the etiology of these unique risk factors between ESCC and EAC will help to explore the reasons for gender differences. In contrast, women (AAPC: ‐6.2%) have a faster decline in esophageal cancer mortality than men (AAPC: ‐3.5%). In recent years, the progress of neoadjuvant chemotherapy may be related to the difference in trends between men and women. Compared with men, women showed excellent pathological results after receiving preoperative neoadjuvant chemotherapy (women to men: 66.7% vs. 36.1%), but the possibility of postoperative complications was less (females vs males: 28.6% vs 52.8%).^22^

Compared with the urban area, as shown in Figure 1, The morbidity and mortality in rural areas are much higher. Some previous studies have reached similar conclusions.^18^, ^23^, ^24^ It was found that the cost of health care, income level, education level, and family gathering of high‐risk groups also contributed. First, patients with esophageal cancer in rural areas have always experienced delayed diagnosis and treatment. The high mortality rate may be related to poor economic capacity and insufficient medical resources.^25^ A survey conducted in seven cities/countries including Linzhou and Cixian showed that the annual cost of illness (ACI) for urban patients with esophageal cancer was US$13,029, while the ACI for rural patients was only US$3,504.^26^ Second, there is a huge gap in income levels between urban and rural areas. The relatively low quality of life in rural areas will undoubtedly have a negative impact on the occurrence of diseases. According to the National Bureau of Statistics, from 2002 to 2014, the per capita disposable income of urban residents increased from 7702.8 yuan to 28,843.9 yuan, while the per capita disposable income of rural residents increased from 2475.6 yuan to 10 488.9 yuan.^27^ Third, receiving a high level of education is associated with an improved survival rate of patients receiving curative treatment (HR: 0.82, 95% CI: 0.69, 0.99).^28^ But in the past two decades, higher education opportunities in rural areas have always lagged behind those in cities.^29^ In addition, due to the lack of education in rural areas, insufficient awareness of disease prevention is also a reason. Fourth, among the high‐risk populations in Henan Province, patients with familial esophageal cancer have some similar characteristics, that is, several generations of these family members live together in rural areas with limited mobility.^30^ Finally, the higher smoking rate in rural areas (62% vs 44%) may also be one of the potential reasons for the higher incidence of esophageal cancer in rural areas.

Although the incidence and mortality of esophageal cancer have been declining in some areas in recent years, the survival rate of patients is still very low due to its extremely aggressive nature.^1^ By pooling and analyzing the survival data of Chinese cancer patients from 2003 to 2015, the study found that the age‐standardized 5‐year relative survival rate of esophageal cancer has steadily increased in the past decade (average rate of change: 2.9%, 95% CI : 0.7%, 5.2%). However, during 2012‐2015, the 5‐year relative survival rate for esophageal cancer was only 30.3% (95% CI: 29.6%, 31.0%).^31^

## 5 CONCLUSION

To sum up, from 2005 to 2015, the incidence of esophageal cancer in China increased first, then decreased, and the mortality rate gradually decreased. The incidence and mortality of esophageal cancer in men are higher than women, and in rural areas than in urban areas.

It suggests that the existing diagnosis and treatment methods can be maintained. On the other hand, timely intervention measures should be taken to the known risk factors of esophageal cancer. And more research should be conducted to explore the underlying reasons (such as the molecular mechanism of genetic influence) that affect the changes in the incidence and mortality of esophageal cancer.

## Conflict of interest

The author did not disclose.

## Author contribution

Ping Zhiguang conceived and designed this research. He Feifan, Qin Xiaoyue, Wan Zhanyong and Li Wei arranged and analyzed the data. He Feifan and Wang Junyi explained data and drafted manuscripts, tables and figures. Liu Li and Ping Zhiguang reviewed the manuscript.

## Data Availability

All raw data are publicly available through the China Central Cancer Registry.

## References

[1] Arnal MJD , Arenas ÁF , Arbeloa ÁL . Esophageal cancer: Risk factors, screening and endoscopic treatment in Western and Eastern countries. World J Gastroenterol. 2015;21(26):7933. 26185366 10.3748/wjg.v21.i26.7933 PMC4499337

[2] He J , Chen W . 2018 Chinese Cancer Registry Annual Report. Beijing: People's Medical Publishing House; 2019.

[3] Bray F , Ferlay J , Soerjomataram I , Siegel RL , Torre LA , Jemal A . Global cancer statistics 2018: GLOBOCAN estimates of incidence and mortality worldwide for 36 cancers in 185 countries. CA Cancer J Clin. 2018;68(6):394‐424. 30207593 10.3322/caac.21492

[4] Lin Y , Totsuka Y , He Y , Kikuchi S , Qiao Y , Ueda J , et al. Epidemiology of esophageal cancer in Japan and China. J Epidemiol. 2013:JE20120162. 10.2188/jea.JE20120162 PMC3709543 23629646

[5] Shin A , Won YJ , Jung HK , Kong HJ , Jung KW , Oh CM , et al. Trends in incidence and survival of esophageal cancer in Korea: analysis of the Korea central cancer registry database. J Gastroenterol Hepatol. 2018;33(12):1961‐1968. 29802647 10.1111/jgh.14289 PMC6334276

[6] Patel N , Benipal B . The incidence of esophageal cancer in the United States from 2001 to 2015: an analysis of cancer statistics in 50 states in the United States. Cureus. 2018;10(12). 10.7759/cureus.3709 PMC6373890 30788198

[7] Lim D , Ha M , Song I . The mortality trend of major cancers in Korea from 1983 to 2012, and a joint analysis was conducted. Cancer epidemic. 2015;39(6):939‐ 946. 10.1016/j.canep.2015.10.023 26523983

[8] Eun, SJ . Trends in mortality from road traffic injuries in South Korea, 1983–2017: Joinpoint regression and age‐period‐cohort analyses. Accident Analysis & Prevention. 2020;134:105325‐105325 31706185 10.1016/j.aap.2019.105325

[9] Amorim CA , Perrota de Souza L , Moreira JP , Luiz RR , Carneiro AJdV , De Souza HS . Geographic distribution and time trends of esophageal cancer in Brazil from 2005 to 2015. Molecular and clinical oncology. 2019;10(6):631‐638. 31086670 10.3892/mco.2019.1842 PMC6488946

[10] Ilic M , Kocic S , Radovanovic D , Macuzic IZ , Ilic l . Trend in esophageal cancer mortality in Serbia, 1991‐2015 (a population‐based study): an age‐period‐cohort analysis and a joinpoint regression analysis.: official journal of the Balkan Union of Oncology. 2019;24(3):1233‐9. Journal of BUON. 2019;24(3):1233‐1239. 31424684

[11] Dong Jing , Thrift Aaron P . Alcohol, smoking and risk of oesophago‐gastric cancer. Best Practice & Research Clinical Gastroenterology. 2017;31(5):509‐517. 29195670 10.1016/j.bpg.2017.09.002

[12] Song Y , Zhao L , Palipudi KM , Asma S , Morton J , Talley B , et al. Tracking MPOWER in 14 countries: results from the Global Adult Tobacco Survey, 2008–2010. Global health promotion. 2016; 23(2_suppl):24‐37. 24042973 10.1177/1757975913501911

[13] Decision of the Standing Committee of the National People's Congress on ratifying the WHO Framework Convention on Tobacco Control 2005 [Available from:http://www.gov.cn/gongbao/content/2005/content_77784.htm

[14] Decree of the Ministry of Health of the People's Republic of China 2011[Available from: http://www.gov.cn/gongbao/content/2011/content_1955012.htm

[15] Nan Y , Xi Z , Yang Y , Wang L , Tu M , Wang J , et al. The 2015 China Adult Tobacco Survey: exposure to second‐hand smoke among adults aged 15 and above and their support to policy on banning smoking in public places. Chinses Journal of Epidemiology. 2016; 37(6):810‐815. 10.3760/cma.j.issn.0254-6450.2016.06.014 27346107

[16] Liang T . On the Meaning and Content of Health Care Investment in China' rural Areas under the Economic Crisis—analysis based on the demography perspective. Journal of Shandong Academy of Governance. 2009；2009(3):72‐74.

[17] Wang M , Hao C , Xie S , Ma S , Ma Q , Zheng R , et al. Efficacy of endoscopic treatment on patients with severe dysplasia/carcinoma in situ of esophageal squamous cell carcinoma: A prospective cohort study. Chinese Journal of Cancer Research. 2019; 31(2):357‐365. 31156306 10.21147/j.issn.1000-9604.2019.02.10 PMC6513741

[18] He Y , Li D , Shan B , Liang D , Shi J , Chen W , et al. Incidence and mortality of esophagus cancer in China, 2008− 2012. Chinese Journal of Cancer Research. 2019; 31(3):426‐434. 31354211 10.21147/j.issn.1000-9604.2019.03.04 PMC6613512

[19] Lao‐Sirieix P , Fitzgerald RC . Screening for oesophageal cancer. Nature reviews Clinical oncology. 2012; 9(5):278‐287. 10.1038/nrclinonc.2012.35 22430857

[20] Wang G‐Q , Jiao G‐G , Chang F‐B , Fang W‐H , Song J‐X , Lu N , et al. Long‐term results of operation for 420 patients with early squamous cell esophageal carcinoma discovered by screening. The Annals of thoracic surgery. 2004; 77(5):1740‐1744. 15111177 10.1016/j.athoracsur.2003.10.098

[21] Kort EJ , Sevensma E , Fitzgerald TL . Trends in esophageal cancer and body mass index by race and gender in the state of Michigan. BMC gastroenterology. 2009；9(1)：47. 19545449 10.1186/1471-230X-9-47 PMC2708181

[22] Nishino T , Yoshida T , Inoue S , Fujiwara S , Goto M , Minato T , et al. Gender differences in clinicopathological features and prognosis of squamous cell carcinoma of the esophagus. Esophagus. 2017; 14(2):122‐130.

[23] Tang W‐R , Fang J‐Y , Wu K‐S , Shi X‐J , Luo J‐Y , Lin K . Epidemiological characteristics and mortality prediction of esophageal cancer in China from 1991 to 2012. Asian Pac J before cancer. 2014;15 (16): 6929‐ 6934. 10.7314/apjcp.2014.15.16.6929 25169548

[24] Zhang S , Zhang M , Li G , Wei W , Meng F , Liu Z , et al. Analysis of the incidence and mortality of esophageal cancer in China from 2003 to 2007. Chinese cancer. 2012;21(4): 241‐ 247.

[25] Zeng H , Zheng R , Guo Y , Zhang S , Zou X , Wang N , et al. Cancer survival rate in China from 2003 to 2005: a population‐based study. International J Cancer. 2015;136(8):1921‐1930. 10.1002/ijc.29227 25242378

[26] Yang Z , Zeng H , Xia R , Liu Q , Sun K , Zheng R , et al. Annual disease costs of gastric and esophageal cancer patients in urban and rural China: a multicenter study. Chinese Cancer Research. 2018;30(4): 439. 10.21147/j.issn.1000-9604.2018.04.07 PMC6129568 30210224

[27] Shoujia H , Song X , Zhao X , Shuang L , Cheng R , Chen P , et al. Comparative analysis of the survival rate of patients with esophageal squamous cell carcinoma in urban and rural areas. Chinese Journal of Oncology. 2017;44(15): 773‐777.

[28] Linde G , Sandin F , Johnson J , Lindblad M , Lendl L , Hedberg J . The patient’s education level affects the treatment allocation and prognosis of esophagus and gastroesophageal junction cancer in Sweden. Cancer epidemic. 2018;52: 91‐98. 10.1016/j.canep.2017.12.008 29278841

[29] Wu W . The latest evolution of China's higher education urban‐rural inequality based on CGSS2015 data. Journal of China Agricultural University (Social Science Edition). 2019;5: 13.

[30] Li C , Li P , Wu H , Guo T , Zhang Y , Shao S , et al. Epidemiological survey of esophageal cancer in the population with high incidence in Henan Province. J Science B of Zhejiang University. 2006;(1): 10.

[31] Zeng H , Chen W , Zheng R , Zhang S , Ji JS , Zou X , et al. Changes in cancer survival rates in China during 2003‐15: a pooled analysis of 17 population‐based cancer registries. The Lancet Global Health. 2018;6(5): e555‐e567. 29653628 10.1016/S2214-109X(18)30127-X

